# White cell count in the normal range and short-term and long-term mortality: international comparisons of electronic health record cohorts in England and New Zealand

**DOI:** 10.1136/bmjopen-2016-013100

**Published:** 2017-02-17

**Authors:** Anoop Dinesh Shah, Simon Thornley, Sheng-Chia Chung, Spiros Denaxas, Rod Jackson, Harry Hemingway

**Affiliations:** 1 Farr Institute of Health Informatics Research, UCL Institute of Health Informatics, London, UK; 2 University College London Hospitals NHS Trust, London, UK; 3 Counties Manukau District Health Board, Auckland, New Zealand; 4 Faculty of Medical and Health Sciences, University of Auckland, Auckland, New Zealand

**Keywords:** Mortality, Leukocyte count, Electronic health records, Cohort studies

## Abstract

**Objectives:**

Electronic health records offer the opportunity to discover new clinical implications for established blood tests, but international comparisons have been lacking. We tested the association of total white cell count (WBC) with all-cause mortality in England and New Zealand.

**Setting:**

Primary care practices in England (ClinicAl research using LInked Bespoke studies and Electronic health Records (CALIBER)) and New Zealand (PREDICT).

**Design:**

Analysis of linked electronic health record data sets: CALIBER (primary care, hospitalisation, mortality and acute coronary syndrome registry) and PREDICT (cardiovascular risk assessments in primary care, hospitalisations, mortality, dispensed medication and laboratory results).

**Participants:**

People aged 30–75 years with no prior cardiovascular disease (CALIBER: N=686 475, 92.0% white; PREDICT: N=194 513, 53.5% European, 14.7% Pacific, 13.4% Maori), followed until death, transfer out of practice (in CALIBER) or study end.

**Primary outcome measure:**

HRs for mortality were estimated using Cox models adjusted for age, sex, smoking, diabetes, systolic blood pressure, ethnicity and total:high-density lipoprotein (HDL) cholesterol ratio.

**Results:**

We found ‘J’-shaped associations between WBC and mortality; the second quintile was associated with lowest risk in both cohorts. High WBC within the reference range (8.65–10.05×10^9^/L) was associated with significantly increased mortality compared to the middle quintile (6.25–7.25×10^9^/L); adjusted HR 1.51 (95% CI 1.43 to 1.59) in CALIBER and 1.33 (95% CI 1.06 to 1.65) in PREDICT. WBC outside the reference range was associated with even greater mortality. The association was stronger over the first 6 months of follow-up, but similar across ethnic groups.

**Conclusions:**

Clinically recorded WBC within the range considered ‘normal’ is associated with mortality in ethnically different populations from two countries, particularly within the first 6 months. Large-scale international comparisons of electronic health record cohorts might yield new insights from widely performed clinical tests.

**Trial registration number:**

NCT02014610.

**Strengths and limitations of this study**

- The main strength of this study is that we showed similar associations of total white cell count with mortality in two ethnically different populations from different countries.
- Both cohorts were large and population-based, avoiding the selection bias inherent in bespoke cohorts with low response rates.
- A limitation of this study is that white cell count and other covariates were measured only when thought to be clinically necessary, so some covariate data were missing.
- As this is an observational study it can be used to infer association but not causation, and residual confounding may partly account for the observed association between total white cell count and mortality.

## Introduction

A fundamental question in clinical medicine is ‘what does this blood test result mean?’ Relevant evidence in answering this question may come from examining the prognostic significance of blood tests recorded in usual clinical care. The direct clinical applicability, large sample sizes and population base of electronic health record cohorts, which are increasingly available for research in different countries, might provide opportunities to discover and replicate associations between clinically recorded measurements and patient outcomes. However, to date there have been few international comparisons of the prognostic validity of blood tests performed in primary care, partly because of the challenge of accessing such data, and harmonising the structure and coding of electronic healthcare records between countries. Nevertheless, such international comparisons might help to evaluate the robustness of associations among patients with different ethnic backgrounds and different profiles of risk.

We chose to compare England and New Zealand because they have different healthcare systems and ethnically different populations (the English population is predominantly Caucasian, with small proportions of South Asian or black ethnicities^1^ whereas New Zealand contains a sizeable proportion of Māori, Pacific, Chinese and South Asian people^2^). However, both countries have pseudonymised primary care data linked with other data sources available for research on a large scale, making this type of study feasible. The data in both countries includes laboratory values, medication, cardiovascular risk factors and death registrations (see online supplementary table S1).

10.1136/bmjopen-2016-013100.supp1 supplementary data

We studied one of the most widely performed blood tests in primary care, the total white cell count. Despite the ubiquity of this test, it is unclear how strongly a clinically recorded ‘normal’ value in the general population is associated with subsequent short-term and long-term mortality. White cell counts vary between ethnic groups^3^ and are associated with inflammation, smoking, obesity and high systolic blood pressure.^4^ Previous bespoke cohort studies with white cell counts measured under research conditions suggest a link between high white cell count and increased risk of coronary disease^5^ ^6^ and long-term mortality (see online supplementary table S2).^7–17^ The largest previous study involved 438 500 government employees and their families in South Korea and accrued 48 757 events,^7^ but the largest study to compare ethnic groups was much smaller, with only 1062 events (Reasons for Geographical and Racial Differences in Stroke (REGARDS) study).^8^ As far as we are aware, there are no general population studies of white cell counts and mortality that used clinical rather than research measures of white cell counts. However, it is important to know the prognostic significance of white cell counts measured for diverse indications in usual clinical care. White cell count even within the normal range can be affected by a range of chronic and acute illnesses^18^ which prompts the question, not answered in previous studies, as to whether it is more strongly associated with short-term than long-term prognosis.

Our objectives were (1) to use clinically recorded white cell counts in diverse populations to replicate previous observations of the association of white cell count with mortality and (2) to extend these observations by comparing short-term and long-term associations, and investigating interactions with ethnicity, age, sex and smoking. This study was a new collaboration between the CALIBER (ClinicAl research using LInked Bespoke studies and Electronic health Records) programme in England^19^ (primary care data linked to hospitalisation, mortality and acute coronary syndrome registries) and PREDICT in New Zealand^20^ (cardiovascular risk assessments from primary care linked to hospital admissions, mortality, dispensed medication and laboratory results).

## Methods

We carried out a cohort study using information recorded in usual clinical care in electronic health records in England and New Zealand. Characteristics of data sources analysed in two countries are summarised in online supplementary table S1.

### CALIBER study population (England)

The study population was drawn from the CALIBER,^19^ which links four sources of electronic health data in England: primary care health records (coded diagnoses, clinical measurements, laboratory results and prescribed medication) from general practices contributing to the Clinical Practice Research Datalink (CPRD),^21^ coded hospital discharges (Hospital Episode Statistics, HES), the Myocardial Ischaemia National Audit Project (MINAP)^22^ and death registrations. CALIBER contains data from 244 general practices which consented to the data linkage; these practices contained 3.9% of the population of England in 2006. The linkage was carried out in October 2010 by a trusted third party, using a deterministic match between National Health Service number, date of birth and sex. CALIBER studies have demonstrated associations of age, sex,^23^ blood pressure,^24^ neutrophil, eosinophil and lymphocyte counts,^25^ ^26^ and type 2 diabetes^27^ with initial presentation of cardiovascular diseases.

The study period was January 1997 to March 2010, and patients were eligible for inclusion when they had been registered for at least 1 year with a practice meeting research data recording standards.

### PREDICT study population (New Zealand)

In New Zealand, approximately one-third of general practitioners use PREDICT, a web-based clinical decision support application to assess cardiovascular risk for primary prevention, and the PREDICT software captures this information centrally including commonly measured risk factors for cardiovascular disease (smoking status, diabetes status, gender, age, systolic blood pressure).^20^ ^28^ These risk assessment records are linked to national databases of hospital admission data and mortality using an encrypted New Zealand National Health Index (NHI) number. Records were also linked with the New Zealand Pharmaceutical Information database,^29^ a national register of community dispensing, and TestSafe, a repository of laboratory test results for the Auckland and Northland region of the North Island of New Zealand. TestSafe contains community and hospital laboratory results from July 2006 onwards; prior to this date only hospital test results were available from this source, or community tests which were also copied to hospital services. White cell count results were linked to PREDICT information using the encrypted NHI. This study includes PREDICT patients assessed between 1 July 2005 and 24 July 2012, as full dispensing, laboratory and outcome data were available for this period.

### Ethical considerations

All data sources were pseudonymised and researchers did not have access to direct patient identifiers. This CALIBER study is registered on clinicaltrials.gov (NCT02014610, https://clinicaltrials.gov/ct2/show/results/NCT02014610).

### Inclusion and exclusion criteria

In both countries, patients aged 30–75 years with no prior history of cardiovascular disease (coronary artery disease, cardiac arrest, ischaemic or haemorrhagic stroke, transient ischaemic attack, abdominal aortic aneurysm or peripheral arterial disease) and no use of loop diuretics in the previous 6 months were eligible. In PREDICT, prior cardiovascular disease was ascertained by the cardiovascular risk assessment questionnaire and hospitalisation records; in CALIBER prior cardiovascular disease was identified in primary care (Read codes for diagnoses) or hospitalisation records (International Classification of Diseases, Tenth Revision (ICD-10) codes) or an entry in the acute coronary syndrome registry. The phenotyping algorithms for CALIBER have been described in detailed in previous studies^19^ ^23–27^ and are available on the CALIBER data portal (http://www.caliberresearch.org/portal).

In CALIBER, patients entered the study on the date of the first white cell count measurement after study eligibility. In PREDICT, patients entered the study on the date of the cardiovascular risk assessment. The most recent measure of total white cell count up to 5 years before or 2 weeks after cardiovascular risk assessment was chosen. If no white cell count was available during this period, it was considered missing.

### Outcomes

The primary outcome of the study was all-cause mortality. We identified patients who had died by linkage to the relevant national death registry, with follow-up in New Zealand until 26 July 2012 and in CALIBER until 25 March 2010.

### Other covariates

Cardiovascular risk factor information such as smoking status, blood pressure and total:high-density lipoprotein (HDL) cholesterol ratio were collected in PREDICT as part of the cardiovascular risk assessment and were largely complete. In CALIBER, we derived smoking status (current smoker or non-smoker, to match the PREDICT categories) using primary care records prior to study entry, and extracted the most recent value of continuous risk factors (blood pressure, total cholesterol and HDL cholesterol) up to 1 year prior to study entry.

Total white cell counts can be affected by factors such as infections, autoimmune diseases, medication and haematological conditions. Similar to our recent CALIBER studies on differential white cell counts,^25^ ^26^ we sought to differentiate between a patient's long-term ‘stable’ white cell count, and values obtained when the patient had an ‘acute’ condition which may alter white cell counts. We adapted a set of validated criteria published by the eMERGE consortium^30^ (electronic Medical Records and Genomics) for studying genetic determinants of the stable white cell counts, which takes into account cancer diagnoses, haematological diagnoses, use of steroids or immune modulating medication, recent vaccination and recent symptoms or diagnoses of infection. We used prescription, symptom, diagnosis and hospitalisation data in the primary care and secondary care records in CALIBER to assess whether the patient was clinically ‘acute’ or ‘stable’ at the time of the blood test; see online supplementary material methods for more details.

Diabetes status was assessed in CALIBER by a diagnosis of diabetes recorded prior to study entry in primary care (as a Read code) or in a hospital admission (as an ICD-10 code in Hospital Episode Statistics).^27^ In PREDICT, diabetes status was entered by the general practitioner into the web-based cardiovascular risk assessment form. In addition, we considered patients to be diabetic if they had been dispensed an oral hypoglycaemic agent or insulin in the 6 months before assessment, or if they had been hospitalised with a primary diagnosis of diabetes within the previous 5 years.

New Zealand ethnicity data were recorded in PREDICT and national data sources, and were classified according to a standardised protocol,^31^ which prioritises Māori then Pacific ethnic groups if more than one ethnic group is recorded. Individuals were coded as Māori, Pacific, Indian or other. ‘Others’ were mainly of New Zealand European descent. In CALIBER, we classified ethnicity as white, black, South Asian (comprising Indian, Pakistani or Bangladeshi) or ‘other’, according to ethnicity recorded in primary care or during a hospital admission.^1^

### Statistical analysis

We performed analyses on CALIBER and PREDICT data separately using a similar protocol. The primary analysis used a Cox proportional hazards survival model. Individuals were considered censored if they reached the end of the study period alive (PREDICT) or transferred out of the practice (CALIBER). The white cell counts were grouped into quintiles based on the distribution of observed values in the CALIBER data, in order to avoid assuming any particular shape for the association. We split the top and bottom quintiles into values within and outside the reference range to assess whether any associations observed were confined to extreme values. As there is no consensus on reference ranges in the literature, we used the reference range of the laboratory of our local university hospital, which was 3–10×10^9^ cells/L.

We assessed the proportional hazards assumption by plotting the scaled Schoenfeld residuals against log time.^32^ Survival models included the following cardiovascular risk factors, chosen a priori: age, sex, total:HDL cholesterol ratio, systolic blood pressure, diabetes status and current smoking status.

All analyses were performed using R software (V.3) (R Development Core Team. R: A language and environment for statistical computing. Vienna: R Foundation for Statistical Computing, 2007.) using the *survival* (Therneau T. A Package for Survival Analysis in S. R package version 2.37–7, 2014. http://CRAN.R-project.org/package=survival) package for Cox regression. We handled missing covariate data using multiple imputation, with 10 multiply imputed data sets, generated using the *mice*^33^ and *CALIBERrfimpute*^34^ R packages (see online supplementary methods). Supporting analyses included assessment for interactions with age group, smoking status, sex, ethnicity and whether the total white cell count was measured when the patient was clinically stable.

## Results

### Comparison of England and New Zealand populations

**Figure 1:**
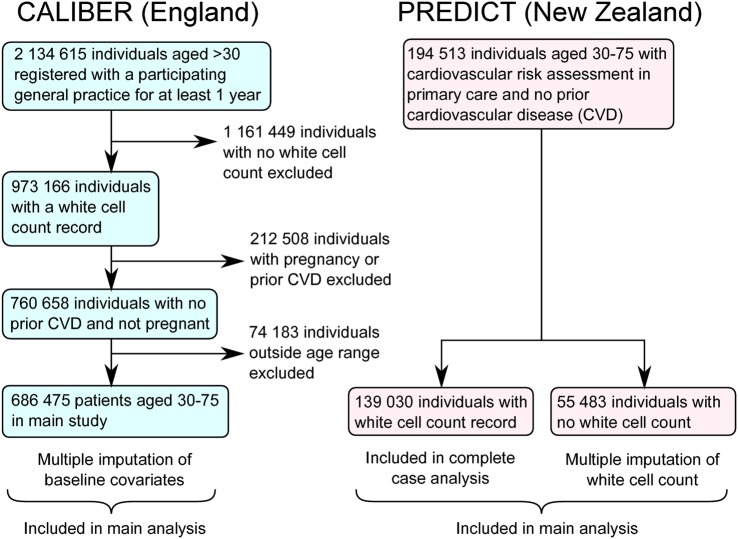
Patient flow diagrams for CALIBER and PREDICT studies. CALIBER, ClinicAl research using LInked Bespoke studies and Electronic health Records.

We analysed 686 475 individuals in CALIBER and 194 513 individuals in PREDICT (figure 1). The median age was 50 years in CALIBER and 55 years in PREDICT, and 45% were men (table 1). There were marked differences in ethnicity: the majority of patients in CALIBER were white (92% of those with ethnicity recorded, 383 428 out of 416 828), but in PREDICT just over half were European (104 000/194 513, 53%), and significant proportions of individuals belonged to Asian, Indian, Pacific or Māori ethnic groups. There were also differences between England and New Zealand in major risk factors for mortality: the prevalence of smoking was higher in England (24.2% vs 16.4%) but diabetes was more prevalent in the New Zealand cohort (4.2% and 8.6%). In PREDICT, 139 030 individuals (71%) had at least one white cell count recorded, and 77% (107 063/109 874) of these records were taken within 1 year before or 2 weeks after risk assessment. All patients in CALIBER had a record of a white cell count (as it was one of the inclusion criteria) (table 1). Patients in CALIBER tended to be younger than those in PREDICT (median age 50 vs 55) and were less likely to be diabetic (4.2% vs 8.6%, p<0.001), but more likely to smoke (24% vs 16%, p<0.001) (table 1).

**Table 1:** Study population by gender and country

	CALIBER (England)			PREDICT (New Zealand)		
Characteristics	Women	Men	Overall	Women	Men	Overall
N patients	401 997	284 478	686 475	86 084	108 429	194 513
Age in years, median (IQR)	49 (39, 60)	52 (42, 61)	50 (40, 60)	57 (50, 63)	52 (46, 60)	55 (47, 62)
N (%) with white cell count record*	401 997 (100%)	284 478 (100%)	686 475 (100%)	63 880 (74.2%)	75 150 (69.3%)	139 030 (71.5%)
White cell count (×10^9^/L), median (IQR)	6.7 (5.5, 8.1)	6.6 (5.5, 8.0)	6.6 (5.5, 8.1)	6.6 (5.4, 8.0)	6.7 (5.6, 8.1)	6.6 (5.5, 8.0)
Ethnicity						
N (%) with ethnicity recorded	256 726 (63.9%)	160 102 (56.3%)	416 828 (60.7%)	86 084 (100%)	108 429 (100%)	194 513 (100%)
White (CALIBER)/European (PREDICT)	235 140 (91.6%)	148 288 (92.6%)	383 428 (92.0%)	45 462 (52.8%)	58 538 (54.0%)	104 000 (53.5%)
South Asian (CALIBER)/Indian (PREDICT)	8140 (3.2%)	4810 (3.0%)	12 950 (3.1%)	6811 (7.9%)	9506 (8.8%)	16 317 (8.4%)
Pacific (PREDICT)	–	–	–	12 810 (14.9%)	15 754 (14.5%)	28 564 (14.7%)
Māori (PREDICT)	–	–	–	12 193 (14.2%)	13 777 (12.7%)	25 970 (13.4%)
Asian (PREDICT)	–	–	–	7306 (8.5%)	8570 (7.9%)	15 876 (8.2%)
Black (CALIBER)	6373 (2.5%)	3261 (2.0%)	9634 (2.3%)	–	–	–
Other	7073 (2.8%)	3743 (2.3%)	10 816 (2.6%)	1502 (1.7%)	2284 (2.1%)	3786 (2.0%)
Current smoker, n (%)†	87 540/385 575 (22.7%)	74 003/268 766 (27.5%)	161 543/654 341 (24.7%)	12 275/86 084 (14.3%)	19 518/108 429 (18.0%)	31 793/194 513 (16.4%)
Systolic blood pressure in mm Hg, median (IQR)†	130 (119, 144)	140 (128, 150)	134 (120, 148)	130 (120, 140)	130 (120, 140)	130 (120, 140)
Total:HDL cholesterol ratio, median (IQR)†	3.6 (2.9, 4.5)	4.4 (3.5, 5.3)	4.0 (3.2, 4.9)	3.6 (2.9, 4.4)	4.3 (3.5, 5.2)	4.0 (3.2, 4.9)
Diabetes at baseline, n (%)	12 741 (3.2%)	16 219 (5.7%)	28 960 (4.2%)	7764 (9.0%)	8882 (8.2%)	16 646 (8.6%)
Deaths during follow-up, n (%)	9636 (2.4%)	9961 (3.5%)	19 597 (2.9%)	892 (1.0%)	1338 (1.2%)	2230 (1.1%)
Follow-up time (years), median (IQR)	4.21 (1.96, 6.42)	3.75 (1.73, 5.97)	4.01 (1.86, 6.23)	2.23 (0.98, 3.78)	2.21 (0.99, 3.86)	2.22 (0.99, 3.83)
Year of enrolment, %						
1998–2004	48.6%	42.6%	46.1%	0	0	0
2005–2008	40.9%	45.1%	42.6%	40.5%	31.7%	36.0%
2009–2010	10.6%	12.3%	11.3%	28.2%	43.9%	36.3%
2011–2012	0	0	0	31.4%	24.4%	27.8%

*In PREDICT, we used the most recent total white cell count within 5 years prior to 2 weeks after the cardiovascular risk assessment. In CALIBER, the study start date was the date of the white cell count measurement, and patients without any white cell count measurement were excluded.

†In CALIBER, we used the most recent blood pressure and cholesterol measurements within 1 year prior to study entry. Blood pressure was available in 63.8% of people, total cholesterol in 32.0% and smoking status in 95.3%. In PREDICT, these measurements were taken at the time of the cardiovascular risk assessment, and were completely recorded.

CALIBER, ClinicAl research using LInked Bespoke studies and Electronic health Records; HDL, high-density lipoprotein.

In the PREDICT cohort, factors associated with non-recording of white cell count included younger age, current smoking, lower total:HDL cholesterol ratio, higher systolic blood pressure, male gender and Māori ethnicity (see online supplementary table S3).

### Correlates of white blood cell count in England and New Zealand

Lower white cell count was associated with Asian or European ethnicity in PREDICT and black ethnicity in CALIBER, whereas higher white cell count was associated with South Asian ethnicity in CALIBER, and Māori or Pacific ethnicity in PREDICT. Higher white cell count was associated with current smoking, diabetes and higher total:HDL cholesterol ratio in both countries (table 2).

**Table 2:** Characteristics of study populations by category of white cell count

		Total white cell count category (×10^9^/L)						
			‘Normal’ range					
	Population	<2.95	2.95–5.35	5.35–6.25	6.25–7.25	7.25–8.65	8.65–10.05	≥10.05
N patients	CALIBER	2718	145 968	135 107	141 491	133 816	68 708	58 667
	PREDICT	248	28 908	28 312	29 136	27 466	13 203	11 757
Men, n (%)	CALIBER	906 (33.3%)	58 242 (39.9%)	58 588 (43.4%)	60 565 (42.8%)	55 429 (41.4%)	27 092 (39.4%)	23 656 (40.3%)
	PREDICT	92 (37.1%)	13 955 (48.3%)	15 565 (55.0%)	16 267 (55.8%)	15 362 (55.9%)	7250 (54.9%)	6659 (56.6%)
Age, median (IQR)	CALIBER	50 (40, 60)	51 (41, 60)	51 (41, 61)	51 (41, 61)	49 (40, 60)	48 (38, 59)	46 (37, 58)
	PREDICT	58 (52, 64)	56 (50, 63)	56 (48, 63)	55 (47, 62)	53 (46, 62)	52 (46, 61)	51 (45, 60)
Ethnicity, n (%):								
White	CALIBER	1491 (88.9%)	76 678 (90.3%)	72 833 (91.8%)	78 597 (92.1%)	76 628 (92.4%)	40 658 (92.7%)	36 543 (94.2%)
European	PREDICT	143 (57.7%)	17 041 (58.9%)	15 849 (56.0%)	14 994 (51.5%)	12 934 (47.1%)	5798 (43.9%)	4987 (42.4%)
South Asian	CALIBER	27 (1.6%)	1796 (2.1%)	2321 (2.9%)	2910 (3.4%)	3117 (3.8%)	1682 (3.8%)	1097 (2.8%)
Indian	PREDICT	9 (3.6%)	2003 (6.9%)	2554 (9.0%)	3072 (10.5%)	3206 (11.7%)	1542 (11.7%)	1103 (9.4%)
Asian	PREDICT	47 (19.0%)	4391 (15.2%)	3072 (10.9%)	2556 (8.8%)	1851 (6.7%)	737 (5.6%)	476 (4.0%)
Pacific	PREDICT	17 (6.9%)	2763 (9.6%)	3669 (13.0%)	4681 (16.1%)	5178 (18.9%)	2793 (21.2%)	2788 (23.7%)
Māori	PREDICT	22 (8.9%)	2034 (7.0%)	2531 (8.9%)	3171 (10.9%)	3725 (13.6%)	2074 (15.7%)	2205 (18.8%)
Black	CALIBER	120 (7.2%)	3992 (4.7%)	1965 (2.5%)	1561 (1.8%)	1106 (1.3%)	549 (1.3%)	341 (0.9%)
Current smoker, n (%)	CALIBER	318 (12.3%)	15 691 (11.3%)	20 415 (15.9%)	29 769 (22.1%)	39 694 (31.1%)	27 216 (41.5%)	28 440 (50.9%)
	PREDICT	17 (6.9%)	1644 (5.7%)	2561 (9.1%)	3995 (13.7%)	5423 (19.7%)	3622 (27.4%)	3859 (32.8%)
Diabetes at baseline, n (%)	CALIBER	75 (2.8%)	4307 (3.0%)	4840 (3.6%)	6071 (4.3%)	6710 (5.0%)	3740 (5.4%)	3217 (5.5%)
	PREDICT	14 (5.6%)	1213 (4.2%)	1705 (6.0%)	2470 (8.5%)	3068 (11.2%)	1812 (13.7%)	1803 (15.3%)
Systolic blood pressure, median (IQR)	CALIBER	130 (120, 144)	132 (120, 146)	135 (120, 148)	135 (121, 149)	135 (120, 149)	134 (120, 148)	130 (120, 145)
	PREDICT	125 (112, 138)	130 (120, 140)	130 (120, 140)	130 (120, 140)	130 (120, 140)	130 (120, 140)	130 (120, 140)
Total:HDL cholesterol ratio, median (IQR)	CALIBER	3.5 (2.8, 4.5)	3.7 (3.0, 4.5)	3.9 (3.2, 4.8)	4.1 (3.3, 5.0)	4.2 (3.4, 5.1)	4.3 (3.4, 5.3)	4.4 (3.5, 5.4)
	PREDICT	3.4 (2.8, 4.3)	3.7 (3.0, 4.5)	3.9 (3.2, 4.8)	4.0 (3.3, 4.9)	4.1 (3.3, 5.0)	4.1 (3.4, 5.0)	4.2 (3.4, 5.1)
Acute condition at time of white cell count measurement	CALIBER	925 (34.0%)	25 903 (17.7%)	22 569 (16.7%)	24 737 (17.5%)	25 438 (19.0%)	14 391 (20.9%)	15 842 (27.0%)

CALIBER, ClinicAl research using LInked Bespoke studies and Electronic health Records; HDL, high-density lipoprotein.

### Crude absolute risks of mortality

**Figure 2:**
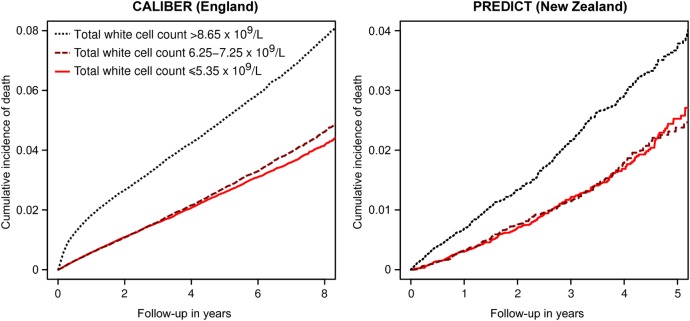
Unadjusted Kaplan-Meier curves for all-cause mortality by total white cell count, in CALIBER and PREDICT. Graphs are shown for top, middle and bottom quintiles of total white cell count. Patients with extreme high or low values are included. To avoid clutter, the second and fourth quintiles are not shown. CALIBER, ClinicAl research using LInked Bespoke studies and Electronic health Records.

Median follow-up was 2.2 years in PREDICT and 4.0 years in CALIBER. The total number of deaths was 2230 in PREDICT and 19 597 in CALIBER. Crude Kaplan-Meier curves showed a greater cumulative incidence of mortality in the highest white cell count quintile (≥ 8.65×10^9^/L) in both populations (figure 2), with a 5-year risk of 5.0% in England and 3.7% in New Zealand. In CALIBER, the gradient of this curve was steepest immediately after study entry, suggesting that the association between white cell count and mortality was particularly strong in the first few months.

### Mortality associations: shape and associations in ‘normal’ range

**Figure 3:**
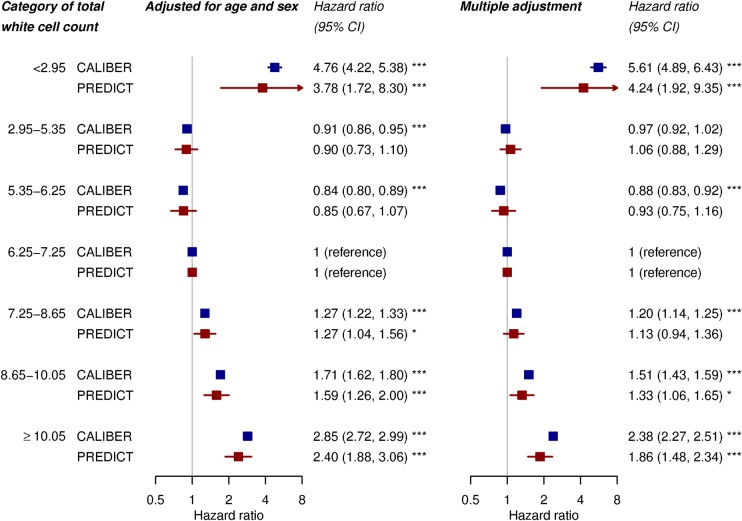
HRs for all-cause mortality by category of total white cell count. Categories are quintiles, with the top and bottom quintiles divided into values within and outside the reference range. ‘Multiple adjustment’ comprised adjustment for age, sex, smoking, diabetes, systolic blood pressure, ethnicity and total:HDL cholesterol ratio. p Values *<0.05, **<0.01, ***<0.001. CALIBER, ClinicAl research using LInked Bespoke studies and Electronic health Records; HDL, high-density lipoprotein.

HRs estimated from Cox models showed a ‘J’-shaped association between white cell count and mortality; the second quintile (5.35–6.25×10^9^/L) was associated with lowest risk. The multiply adjusted HR comparing the highest and middle quintiles in CALIBER was 1.90 (95% CI 1.82 to 1.99). We divided the highest and lowest quintiles into white cell count values within and outside the reference range and found that, compared with the middle quintile, high values within the reference range (8.65–10.05×10^9^/L vs 6.25–7.25×10^9^/L) were associated with significantly increased mortality (adjusted HR 1.51 (95% CI 1.43 to 1.59) in CALIBER and 1.33 (95% CI 1.06 to 1.65) in PREDICT). In comparison, the HR for diabetes in the CALIBER model was 1.41 (95% CI 1.34 to 1.48). For high white cell counts outside the reference range (≥ 10.05×10^9^/L vs 6.25–7.25×10^9^/L) the associations were even stronger (adjusted HR 2.38 (95% CI 2.27 to 2.51) in CALIBER and 1.86 (95% CI 1.48 to 2.34) in PREDICT) (figure 3). Low values of white cell count outside the reference range (<2.95×10^9^/L) were also associated with greater mortality. The associations from multiply adjusted models were slightly attenuated compared with the models adjusted only for age and sex (figure 3).

### Short-term mortality associations

**Figure 4:**
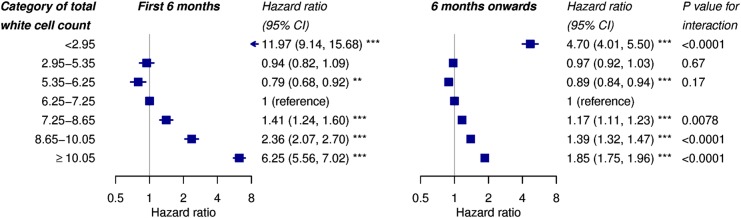
Adjusted HRs for all-cause mortality by category of total white cell count in CALIBER, by time period. Categories are quintiles, with the top and bottom quintiles divided into values within and outside the reference range. HRs were adjusted for age, sex, smoking, diabetes, systolic blood pressure, ethnicity and total:HDL cholesterol ratio. p Values *<0.05, **<0.01, ***<0.001. CALIBER, ClinicAl research using LInked Bespoke studies and Electronic health Records; HDL, high-density lipoprotein.

In the CALIBER cohort, the strength of association was not constant over time; the HR for the highest white cell count quintile decreased with time (Schoenfeld residuals in online supplementary figure S1, p<0.0001 for correlation with log time) (figure 4). We split the follow-up time at 6 months, as there was no statistically significant evidence of non-proportionality of hazards after 6 months. The adjusted HRs for high white cell counts outside the reference range (≥10.05×10^9^/L vs 6.25–7.25×10^9^/L) was 6.25 (95% CI 5.56 to 7.02) for the first 6 months and 1.85 (95% CI 1.75 to 1.96) beyond 6 months.

### Supporting analyses

There was a stronger association of high white cell count with mortality among older people; comparing the highest and middle categories (≥10.05×10^9^/L vs 6.25–7.25×10^9^/L), the adjusted HR in CALIBER was 1.61 (95% CI 1.36 to 1.90) among those aged 30–45 and 2.56 (95% CI 2.40 to 2.72) among those aged 60 or over (see online supplementary figure S2). The association of low white cell count with increased mortality was slightly stronger among current smokers, whereas the association of high white cell count with increased mortality was stronger among ex-smokers or never smokers (see online supplementary figure S3).

About one-fifth of patients in CALIBER (129 805/686 475, 18.9%) were classified as having an ‘acute’ clinical condition on the date of their total white cell count measurement. The most common acute conditions were a diagnosis of infection or symptoms suggestive of infection within the previous month (see online supplementary table S4). The association of ‘stable’ white cell count with mortality was closer to linear within the normal range than that for ‘acute’ white cell count. Low normal white cell count (2.95–5.35×10^9^/L) was associated with greater mortality among acute measurements (HR 1.18, 95% CI 1.09 to 1.28) but lower mortality among stable measurements (HR 0.86, 95% CI 0.81 to 0.92) compared with the middle category (6.25–7.25×10^9^/L) (p for interaction <0.0001) (see online supplementary figure S4). For high normal white cell counts (8.65–10.05×10^9^/L) the association with higher mortality was stronger among stable white cell counts (HR 1.52 compared with 6.25–7.25×10^9^/L, 95% CI 1.43 to 1.63) than acute white cell counts (HR 1.31, 95% CI 1.20 to 1.43; p for interaction 0.0047) (see online supplementary figure S4).

There were no statistically significant interactions with sex (see online supplementary figure S5) or between ethnicities in the English CALIBER data (see online supplementary figure S6). In the New Zealand PREDICT data, the associations were broadly similar across ethnicities, but the HR associated with the highest white cell count was slightly lower for Māori and Pacific peoples than for Europeans (see online supplementary figure S7).

The two methods of multiple imputation used in CALIBER data (normal-based and Random Forest multiple imputation by chained equations (MICE)) yielded almost identical estimates (see online supplementary figure S8). Complete case analysis of PREDICT data yielded similar estimates to the main imputed analysis (see online supplementary figure S9).

## Discussion

As far as the authors are aware, this is the first large-scale population-based assessment of a clinically recorded blood test and mortality across two countries. We found that clinically recorded white cell counts that would currently be considered ‘normal’ are associated with mortality in populations which differ in ethnic composition and risk factors for mortality, and that this association seems particularly strong within the first 6 months.

### Feasibility and value of international comparisons of electronic health record cohorts

We demonstrate the feasibility and value of making international comparisons across heterogeneous primary care electronic health record cohorts. The consistency of associations between the two countries addresses concerns of generalisability (different ethnicities with different baseline white cell counts), selection bias (different selection mechanisms in the two countries) and incomplete recording (different patterns of missing risk factor information in the two countries) and the proliferation of unreplicated research in medicine.^35^ ^36^

### Replication of studies measuring white cell counts under research conditions

It was possible that the cut points used to define high white cell counts in clinical settings (based on the distribution of these measurements in healthy people) might denote a threshold at which a clinically measured risk is associated with higher mortality. This was the case, for example for haemoglobin, where the WHO's thresholds for diagnosis of nutritional anaemia^37^ coincided with the level below which mortality increases in patients with coronary heart disease.^38^ Furthermore, since white cell counts are measured clinically in hundreds of laboratories in England without standardisation, it is possible that measurement issues might dilute any associations. For these reasons it is noteworthy that we replicated a ‘J’-shaped association between clinically measured total white cell count and mortality, consistent with previous studies of white cell counts taken and analysed under standardised research conditions (see online supplementary table S2).^7–17^

Individuals in the second quintile of white cell count (5.35–6.25×10^9^/L) had the lowest mortality risk, and those in the highest quintile had the highest risk (figure 3). At the lower end of the scale, increased risk was restricted to patients with low white cell counts outside the reference range (adjusted HR 5.61 comparing groups <2.95×10^9^/L and 6.25–7.25×10^9^/L in CALIBER). The increased risk of mortality observed in patients with very low white cell counts may be because of malnutrition or comorbidities such as cancer. However, mortality risk was increased at ‘high normal’ levels of total white cell count within the laboratory reference range, that is, at levels which clinicians would typically consider ‘normal’ and would not contribute to clinical decision-making. This association was stronger than the association with diabetes.

A novel finding was that the association of white cell count with mortality was strongest close to the time of white cell count measurement, suggesting that it reflects aspects of the patient's current medical state. However, significant but weaker associations persisted for several years, suggesting that the white cell count is also a useful long-term prognostic marker (figure 4).

Total white cell count is a marker of inflammation, and there are a number of putative biological mechanisms which may explain the inflammation. Air pollution may be one of the causes, as it can cause white cell counts to increase acutely^39^ and is associated with increased incidence of heart failure,^40^ ischaemic heart disease and stroke.^41^ Another potential source of inflammation is periodontitis, which is linked to higher white cell count.^42^ ^43^ The increased risk of mortality observed in patients with very low white cell counts may be because of other comorbidities or malnutrition, which were not recorded in our data set.

### Challenges in international electronic health record studies

Since many countries have national data coded with the ICD it might, at first glance, appear to be straightforward to make international comparisons. But there are many challenges. First, ICD is most widely applied in hospitalisations, rather than in ambulatory care. Second, blood laboratory values (not part of ICD) are much less widely available for research in national samples. Initiatives to foster and promote international collaboration (eg, the Global Alliance for Genomics and Health^44^) may facilitate this. Legal and governance considerations may restrict the sharing of electronic health record data as the patients have not been asked for individual consent, so data may have to be analysed separately in the different countries, as in our study. We were able to access English data locally and New Zealand data remotely. We ran the same scripts to perform a similar analysis but were unable to run a combined analysis on the entire cohort. To address this problem, there is much interest in methodologies for distributed analysis, where the results of analyses at different locations are combined without sharing the raw data.^45^

A key scientific challenge is to harmonise the format of data from different sources, taking into account its provenance. Differences in language, culture, organisation of the healthcare system and availability of data for research may affect the types of data available and their interpretation. For example, in CALIBER we had records of prescribed medication but in PREDICT we had records of dispensed medication. Diagnoses may be recorded using different coding systems (eg, WHO ICD-10 in the UK but ICD-10 Australian Modification in New Zealand). Use of a common terminology (eg, ICD-10, Systematized Nomenclature of Medicine (SNOMED)-CT) would be beneficial. The information models may also be different. Global efforts to standardise the structure of medical records such as openEHR^46^ may help to alleviate this problem in the future, but local knowledge of the healthcare system in each country is essential.

### Strengths

The main strengths of this study were the fact that two cohorts were available in different countries. Both cohorts were large and population-based, avoiding the selection bias inherent in many bespoke cohorts with low response rates.^47^

### Limitations

A limitation of both studies is that white cell count was measured only when it was thought to be clinically necessary, so the probability that a patient has a white cell count depends on a range of factors such as the patient's health-seeking behaviour and the doctor's tolerance of uncertainty or propensity to investigate a patient, as well as the patient's clinical condition. However, we found similar results to previous studies in bespoke cohorts, in which blood testing for white cell count was performed under standardised conditions without any acute or chronic indication (see online supplementary table S2).

Other weaknesses of the study relate to the variation in time from recording of white cell count to cardiovascular assessment (in PREDICT) and the amount of missing data. Complete case analysis assumes there is no selection bias caused by the removal of patients with incomplete records. Multiple imputation relies on the missingness mechanism being ‘missing at random’, that is, whether a value is missing is independent of the value itself conditional on all observed covariates.^33^ ^34^ However, analyses in the two cohorts (with different types of information missing) and analyses using different methods for handling missing data yielded similar results.

Finally, as this is an observational study it can be used to infer association but not causation. Residual confounding may partly account for the observed association between total white cell count and mortality; both may be caused by an underlying chronic inflammatory condition. Nevertheless, clinically a raised white cell count may be useful in the early detection of such conditions, enabling interventions to potentially mitigate their adverse effects.

### Clinical recommendations

It is well known that high white cell counts are associated with acute infections,^4^ and clinicians typically interpret the test in a binary sense—‘normal’ or ‘abnormal’, with normality defined by the laboratory on the basis of the distribution of measurements among ‘healthy’ individuals. We suggest that the white cell count should be considered as a continuous scale, much like blood pressure or cholesterol, for which there are pragmatic treatment thresholds but clinicians understand that the underlying association with risk is continuous. As the total white cell count is a marker of systemic inflammation, we suggest that patients with high normal white cell counts who are apparently asymptomatic should be assessed for potentially modifiable causes of an inflammatory state, such as dental disease.

### Research recommendations

We demonstrate the feasibility and value of international electronic health record collaborations examining blood markers. Future studies should investigate the differential white cell counts (as different white cell subtypes have different functions and determinants in the blood), investigate specific diseases rather than all-cause mortality, and account for acute and chronic conditions at the time of blood sampling. More broadly, we propose that health technology assessment of clinical blood tests should routinely involve large-scale ongoing electronic health record research, to evaluate their associations with mortality and a wide range of morbidity outcomes. We recommend greater use of international replication of new epidemiological findings to verify they are generalisable to populations which differ in ethnic composition and prevalence of risk factors.

## Conclusions

We discovered an association between white cell counts and subsequent mortality, and replicated this finding in a markedly different population from another country. Large-scale international comparisons of cohorts derived from electronic health records might help identify new insights from widely performed clinical tests.
