# Supplementary material for: White cell count in the normal range and short-term and long-term mortality: international comparisons of electronic health record cohorts in England and New Zealand
Source: BMJ Open. 2017 Feb 17;7(2):e013100. doi: 10.1136/bmjopen-2016-013100 (PMC5318564; doi:10.1136/bmjopen-2016-013100)

White cell count in the normal range and short and long term mortality: international comparisons of electronic health record cohorts in England and New Zealand

Supplementary Material

Table of Contents

Supplementary Methods: statistical analysis..2
Acute conditions at time of white cell count measurement: CALIBER................................2
Survival analysis..2
Multiple imputation: CALIBER..2
Multiple imputation: PREDICT..3

Supplementary Tables..4
Supplementary Table S1. Characteristics of CALIBER and PREDICT databases................4
Supplementary Table S2. Previous large studies investigating the association of total white cell count with all-cause mortality in healthy populations...5
Supplementary Table S3. Variables associated with missing white cell count status in a multivariable logistic model in PREDICT...7
Supplementary Table S4. Acute conditions at the time of white cell count measurement in CALIBER...8

Supplementary Figures..9
Supplementary Figure S1. Scaled Schoenfeld residuals for multiply adjusted hazard ratio for mortality comparing quintiles of total white cell count: CALIBER................................9
Supplementary Figure S2. Multiply adjusted hazard ratios for all cause mortality by category of total white cell count, by age group: CALIBER...10
Supplementary Figure S3. Multiply adjusted hazard ratios for all cause mortality by category of total white cell count, by smoking status: CALIBER.......................................11
Supplementary Figure S4. Multiply adjusted hazard ratios for all cause mortality by category of total white cell count, by clinical state: CALIBER...12
Supplementary Figure S5. Multiply adjusted hazard ratios for all cause mortality by category of total white cell count, by sex: CALIBER...13
Supplementary Figure S6. Multiply adjusted hazard ratios for all cause mortality by category of total white cell count, by ethnicity: CALIBER..14
Supplementary Figure S7. Multiply adjusted hazard ratios for all cause mortality by category of total white cell count, by ethnicity: PREDICT...15
Supplementary Figure S8. Multiply adjusted hazard ratios for all cause mortality by category of total white cell count, by method of multiple imputation: CALIBER..............16
Supplementary Figure S9. Hazard ratios for all cause mortality by category of total white cell count for complete case analysis: PREDICT...17

Supplementary Methods: statistical analysis

Acute conditions at time of white cell count measurement: CALIBER

Total white cell counts can be affected by many factors such as infections, autoimmune diseases, medication and haematological conditions. Similar to our recent study on eosinophil counts,[1] we sought to differentiate between a patient's long-term 'stable' total white cell count and results obtained when the patient had an 'acute' condition which may alter leukocyte counts. We used other information in the electronic health record (prescriptions, diagnoses, symptoms, hospitalisations) to assess whether the patient was clinically 'acute' or 'stable' at the time of the blood test, adapting a set of criteria proposed by the eMERGE consortium (electronic Medical Records and Genomics)[2] for studying genetic determinants of the stable leukocyte counts: in hospital on the date of blood test, vaccination in the previous 7 days, anaemia diagnosis within the previous 30 days, symptoms or diagnosis of infection within the previous 30 days, prior diagnosis of myelodysplastic syndrome, prior diagnosis of haemoglobinopathy, cancer chemotherapy or G-CSF within 6 months before index date, the use of drugs affecting the immune system such as methotrexate or steroids within the previous 3 months, prior diagnosis of HIV infection, prior splenectomy or prior dialysis.

Survival analysis

The main analysis used a Cox proportional hazards approach[3] to model the association between white cell count quintiles and time to death, adjusting for the following Framingham risk factors, chosen *a priori*: age, sex, total: HDL cholesterol ratio, systolic blood pressure, diabetes status, and current smoking status. As it was not possible to combine the raw datasets, we used quintiles based on the CALIBER dataset for both CALIBER and PREDICT analyses. In CALIBER, the baseline hazard of the Cox model was stratified by general practice.

All analyses were done using R software (version 3.0.2),[4] using the *survival*[5] package for Cox regression. Restricted cubic splines were used to investigate the relationship between continuous variables and time-to-death as a check on the modelling assumption of linearity. We assessed the proportional hazards assumption by plotting the scaled Schoenfeld residuals against log time.[3] We found that the hazard ratio varied over time (Supplementary Figure S7), so we split the follow-up time into two intervals, pre and post 6 months.

Multiple imputation: CALIBER

In CALIBER, the cohort consisted of individuals with a white cell count record and no prior history of cardiovascular disease, whether or not they had had a formal cardiovascular disease assessment. Total white cell count data was therefore completely observed but some people did not have records of other baseline covariates: total cholesterol, HDL cholesterol, blood pressure or smoking status. We wished to examine for interactions between total white cell count and predictor variables in their association with mortality, so we chose a method of multiple imputation which would account for these interactions. Therefore for the primary analysis we used multiple imputation using chained equations (MICE)[6] with Random Forest multiple imputation.[7]

Random Forest is a machine learning regression method which can automatically account for non-linearities in the associations between predictor variables, and can handle large numbers of variables without encountering problems due to collinearity.[8] It has been shown to perform well on test datasets derived from CALIBER,[8] but can be slow on very large datasets, so we carried out the imputation within general practice, thus also accounting for between-practice variability. As Random Forest multiple imputation is a fairly new method we also performed a secondary analysis using normal-based multiple imputation in MICE. With normal-based MICE we included the general practice as a fixed effect (i.e. a categorical variable) and carried out the imputation separately by age group and sex, which accounted for interactions between these variables without requiring too many additional parameters in the imputation models.

Imputation models included all the variables in the substantive model, event indicator and time in the form of the marginal Nelson-Aalen cumulative hazard, as well as the following auxiliary variables: counts of white cell subtypes (neutrophils, basophils, eosinophils, monocytes and lymphocytes), renal function (estimated glomerular filtration rate), and diastolic blood pressure. We used 10 iterations and generated 10 imputations. We verified that the number of iterations was sufficient by inspecting plots of chain means and variances. We combined the results of analysis using Rubin's rules.

The two methods of multiple imputation used in CALIBER data (normal-based and Random Forest MICE) yielded almost identical estimates (Supplementary Figure S8).

Multiple imputation: PREDICT

The baseline population in PREDICT consisted of all individuals undergoing cardiovascular risk assessment in primary care. Cardiovascular risk factors were almost completely observed but about 30% of people did not have a record of total white cell count. As individuals with a measurement of white cell count would be a (possibly biased) sample, we carried out analyses both limited to those with a white cell count measurement and among the entire baseline population, using multiple imputation to handle missing values. We imputed missing values using Random Forest. Imputation models included all the variables in the substantive model, event indicator and time as the marginal Nelson-Aalen cumulative hazard.[9] We generated 10 imputed datasets and combined the results of analysis using Rubin's rules.

Complete case analysis of PREDICT data yielded similar estimates to the main imputed analysis (Supplementary Figure S9).

References

1. Shah AD, Denaxas S, Nicholas O, Hingorani AD, Hemingway H. Low eosinophil and low lymphocyte counts and the incidence of 12 cardiovascular diseases: a CALIBER cohort study. Open Heart. 2016;3(2):e000477. doi: 10.1136/openhrt-2016-000477
2. Jarvik G, Crosslin D. Group Health Cooperative. White Blood Cell Indices. PheKB; 2012. https://www.phekb.org/phenotype/19
3. Therneau TM, Grambsch PM. Modeling Survival Data: Extending the Cox Model: Springer, 2000.
4. R Development Core Team. R: A language and environment for statistical computing. Vienna: R Foundation for Statistical Computing, 2007.
5. Therneau T. A Package for Survival Analysis in S. R package version 2.37-7, 2014. http://CRAN.R-project.org/package=survival
6. van Buuren S, Groothuis-Oudshoorn K. mice: Multivariate Imputation by Chained Equations in R. J Stat Softw 2011;45(3):1-67. http://www.jstatsoft.org/v45/i03/
7. Shah AD. CALIBERrfimpute: Imputation in MICE using Random Forest. R package version 0.1-6, 2014. http://CRAN.R-project.org/package=CALIBERrfimpute
8. Shah AD, Bartlett JW, Carpenter J, Nicholas O, Hemingway H. Comparison of Random Forest and parametric imputation models when imputing missing data using MICE: a CALIBER study. Am J Epidemiol. 2014;179(6):764-774. doi: 10.1093/aje/kwt312
9. White IR, Royston P. Imputing missing covariate values for the Cox model. Stat. Med. 2009;28(15):1982-98.

Supplementary Tables

Supplementary Table S1. Characteristics of CALIBER and PREDICT databases

	CALIBER (England)	PREDICT (New Zealand)
Data sources		
Full general practice record	All coded data. Some free text available.	No
Cardiovascular risk assessments in primary care	Not systematically recorded	Yes
Laboratory blood tests	Tests requested by GP and reported electronically, and a small number of test results entered from paper reports by the GP before electronic transfer of results was available.	All laboratory tests, including those requested by secondary care
Dispensed medication in the community	No	Yes
Prescribed medication	Yes	No
Hospital admission data	Yes	Yes
Death certificate data	Coded data	Coded data and free text cause of death
Population coverage		
Coverage of country's population	General practices covering about 4% of the English population	Cardiovascular risk assessment from about one-third of general practices, distributed throughout the country; laboratory results for Auckland and Christchurch regions
Number of patients	4.7 million	2 million with laboratory results, 400,000 with cardiovascular risk assessment
Follow-up period	While registered with GP	While resident in New Zealand
Coding		
Primary care diagnoses	Read version 2	Not applicable
Hospital diagnoses	WHO ICD-10	ICD-10 Australian modification
Cause of death	WHO ICD-10	ICD-10 Australian modification
Procedures	Office for Population Censuses and Surveys classification (OPCS)	ICD-10 Australian modification
Time period	GP data from 1990s, hospital data from 2000, cause of death from 2001	2002 onwards

Supplementary Table S2. Previous large studies investigating the association of total white cell count with all-cause mortality in healthy populations

**Author, year, study name**	**Study population**	**N events**	**Clinical WBC measure-ment?**	**Compare ethnic groups?**	**Short and long term risks?**	**Adjusted measure of association with total white cell count**
Jee, 2005, Korean Cancer Prevention Study[1]	438 500 government employees, teachers, and their dependants insured with Korea Medical Insurance Corporation	48 757	-	-	-	HR (95% CI) for WBC ≥9 vs <5 × 10^9/L:<br>Male non-smokers 1.38 (1.22, 1.56)<br>Male smokers 1.14 (1.07, 1.21)<br>Female non-smokers 1.20<br>Female smokers 1.26 (1.12, 1.41)
Margolis, 2005, Women's Health Initiative Observational Study[2]	66 261 women with mean follow-up 6.1 years	1919	-	-	-	HR 1.52 (95% CI 1.33, 1.74) for top vs bottom quartile (P for trend <0.001)
Kabagambe, 2011, REGARDS study[3]	17 845 men and women in the Reasons for Geographic and Racial Differences in Stroke Study cohort	1062	-	Yes	-	HR 1.33 (95% CI 1.03, 1.72) for top vs bottom quartile in Whites (p for trend = 0.03)<br>HR 1.13 (95% CI 0.86, 1.50) for top vs bottom quartile in Blacks (p for trend = 0.55)
Ruggiero, 2007, Baltimore Long-itudinal Study of Aging[4]	2803 people recruited from Baltimore-Washington, DC	944	-	-	-	HR 1.62 (95% CI 0.92, 2.85) for WBC >10 vs 3.5–6.0 × 10^9/L
Tamakoshi, 2007, NIPPON DATA90[5]	6756 Japanese residents followed up from nationwide random survey	576	-	-	-	RR 1.61 (95% CI 1.07, 2.40) for WBC 9–10 × 10^9/L vs 4–4.9 × 10^9/L
Shankar, 2007, Blue Mountains Eye Study[6]	2904 people with no prior cardiovascular disease or cancer	575	-	-	-	RR 1.68 (95% CI 1.35, 2.09) for top vs bottom quartile
Horne, 2015, JUPITER trial[7,8]	17 197 trial participants followed for median 1.9 years	445	-	-	-	HR 1.0 (95% CI 0.85, 1.15) (read from graph) linear trend by tertile comparing adjusted for red cell distribution width, haematocrit, mean corpuscular volume, mean corpuscular haemoglobin concentration, high sensitivity C reactive protein and conventional cardiovascular risk factors

Author, year, study name	Study population	N events	Clinical WBC measure-ment?	Compare ethnic groups?	Short and long term risks?	Adjusted measure of association with total white cell count
De Labry, 1990, Normative Aging Study[9]	2011 healthy men in Boston	183	-	-	-	HR 1.16 (95% CI 1.08, 1.25) per 10^9 higher WBC adjusted for age, cholsterol, systolic blood pressure and smoking
Kim, 2013, Korean elderly cohort[10]	9996 men and women aged 65 or over	118	-	-	-	HR 1.57 (95% 0.88, 2.80) for top vs bottom quartile (P for trend <0.073)
Current study	686 475 people in England (CALIBER) and 194 513 people in New Zealand (PREDICT)	21 827	Yes	Yes	Yes	See results in main paper

CI, confidence interval; HR, hazard ratio; RR, relative risk. Studies are listed in descending order of number of events; results in the table are the maximally adjusted results reported in the paper.

References for studies

1. Jee SH, Park JY, Kim HS, Lee TY, Samet JM. White blood cell count and risk for all-cause, cardiovascular, and cancer mortality in a cohort of Koreans. Am J Epidemiol. 2005;162(11):1062-9.
2. Margolis KL, Manson JE, Greenland P et al. Leukocyte count as a predictor of cardiovascular events and mortality in postmenopausal women: the Women's Health Initiative observational study. Arch Intern Med 2005;165(5):500–508. doi: 10.1001/archinte.165.5.500
3. Kabagambe EK, Judd SE, Howard VJ. Inflammation Biomarkers and Risk of All-Cause Mortality in the Reasons for Geographic and Racial Differences in Stroke Cohort. Am J Epidemiol 2011;174(3):284–292. doi: 10.1093/aje/kwr085
4. Ruggiero C, Metter EJ, Cherubini A, et al. White blood cell count and mortality in the Baltimore Longitudinal Study of Aging. J Am Coll Cardiol 2007;49(18):1841-50.
5. Tamakoshi K, Toyoshima H, Yatsuya H, Matsushita K, Okamura T, Hayakawa T, et al. White blood cell count and risk of all-cause and cardiovascular mortality in nationwide sample of Japanese--results from the NIPPON DATA90. Circ J 2007;71(4):479-85.
6. Shankar A, Mitchell P, Rochtchina E, Wang JJ. The association between circulating white blood cell count, triglyceride level and cardiovascular and all-cause mortality: population-based cohort study. Atherosclerosis 2007;192(1):177-83.
7. Horne BD, Anderson JL, Muhlestein JB et al. Complete blood count risk score and its components, including RDW, are associated with mortality in the JUPITER trial. Eur J Prev Cardiol 2015;22(4):519-526.
8. Ridker PM, Danielson E, Fonseca FAH et al. Rosuvastatin to prevent vascular events in men and women with elevated {C}-reactive protein. N Engl J Med 2001;359(21):2195-2207.
9. de Labry LO, Campion EW, Glynn RJ, Vokonas PS. White blood cell count as a predictor of mortality: results over 18 years from the Normative Aging Study. J Clin Epidemiol 1990;43(2):153-157
10. Kim K-I, Lee J, Heo NJ. Differential white blood cell count and all-cause mortality in the Korean elderly. Exp Gerontol 2013;48(2):103–108. doi: 10.1016/j.exger.2012.11.016

Supplementary Table S3. Variables associated with missing white cell count status in a multivariable logistic model in PREDICT

Total number of patients = 194 498; number with missing white cell count = 55 478

Variable	Comparison		Adjusted odds ratio (95% CI) †
	Low value	High value	
Age at assessment (years) *	45	65	0.80 (0.79, 0.82)
Diabetes	No	Yes	1.02 (0.99, 1.06)
Current smoking status	Non-smoker	Smoker	1.13 (1.10, 1.16)
Total: HDL cholesterol ratio *	2.9	5.3	0.89 (0.88, 0.91)
Systolic blood pressure (mmHg) *	112	146	1.06 (1.04, 1.08)
Gender	Male	Female	0.79 (0.77, 0.80)
Ethnic group	European	Asian	0.46 (0.44, 0.48)
	European	Indian	0.44 (0.42, 0.46)
	European	Maori	1.32 (1.28, 1.36)
	European	Other	0.54 (0.50, 0.59)
	European	Pacific	0.63 (0.61, 0.65)

* For continuous variables, comparisons were taken at the 16th and 84th centiles.

† Adjusted for all variables in the table.

Supplementary Table S4. Acute conditions at the time of white cell count measurement in CALIBER

		Total white cell count category (× 10^9/L)					
		'Normal' range					
	< 2.95	2.95–5.35	5.35–6.25	6.25–7.25	7.25–8.65	8.65–10.05	≥ 10.05
In hospital on date of blood test	60 (2.2%)	292 (0.2%)	249 (0.2%)	331 (0.2%)	377 (0.3%)	335 (0.5%)	732 (1.2%)
Vaccination within previous 7 days	30 (1.1%)	1368 (0.9%)	1293 (1.0%)	1396 (1.0%)	1315 (1.0%)	578 (0.8%)	424 (0.7%)
Anaemia diagnosis within 30 days prior	58 (2.1%)	823 (0.6%)	517 (0.4%)	568 (0.4%)	464 (0.3%)	273 (0.4%)	274 (0.5%)
Infection diagnosis within 30 days prior	262 (9.6%)	8979 (6.2%)	8212 (6.1%)	9111 (6.4%)	9762 (7.3%)	5647 (8.2%)	6380 (10.9%)
Infective symptoms within 30 days prior	194 (7.1%)	6881 (4.7%)	6559 (4.9%)	7317 (5.2%)	7877 (5.9%)	4573 (6.7%)	4909 (8.4%)
Prior diagnosis of myelodysplastic syndrome	19 (0.7%)	60 (0.0%)	19 (0.0%)	27 (0.0%)	21 (0.0%)	7 (0.0%)	9 (0.0%)
Prior diagnosis of haemoglobinopathy	20 (0.7%)	717 (0.5%)	483 (0.4%)	483 (0.3%)	440 (0.3%)	207 (0.3%)	190 (0.3%)
Chemotherapy or G-CSF within 6 months prior	198 (7.3%)	543 (0.4%)	246 (0.2%)	208 (0.1%)	202 (0.2%)	108 (0.2%)	248 (0.4%)
Methotrexate within 3 months prior	5 (0.2%)	436 (0.3%)	446 (0.3%)	501 (0.4%)	559 (0.4%)	381 (0.6%)	337 (0.6%)
Steroid within 3 months prior	61 (2.2%)	1915 (1.3%)	1973 (1.5%)	2755 (1.9%)	3455 (2.6%)	2498 (3.6%)	3719 (6.3%)
Other immune drug within 3 months prior	73 (2.7%)	1878 (1.3%)	1351 (1.0%)	1297 (0.9%)	1311 (1.0%)	731 (1.1%)	723 (1.2%)
Prior diagnosis of cancer	436 (16.0%)	6499 (4.5%)	4836 (3.6%)	4778 (3.4%)	4176 (3.1%)	2089 (3.0%)	2270 (3.9%)
Prior splenectomy	2 (0.1%)	64 (0.0%)	83 (0.1%)	146 (0.1%)	234 (0.2%)	190 (0.3%)	327 (0.6%)
Prior diagnosis of HIV	4 (0.1%)	61 (0.0%)	31 (0.0%)	14 (0.0%)	17 (0.0%)	14 (0.0%)	8 (0.0%)
Prior dialysis	6 (0.2%)	37 (0.0%)	32 (0.0%)	34 (0.0%)	49 (0.0%)	28 (0.0%)	37 (0.1%)
Any acute condition	925 (34.0%)	25903 (17.7%)	22569 (16.7%)	24737 (17.5%)	25438 (19.0%)	14391 (20.9%)	15842 (27.0%)

G-CSF, granulocyte colony stimulating factor; HIV, human immunodeficiency virus

Supplementary Figures

Supplementary Figure S1. Scaled Schoenfeld residuals for multiply adjusted hazard ratio for mortality comparing quintiles of total white cell count: CALIBER

Hazard ratios were adjusted for age, sex, smoking, diabetes, systolic blood pressure, ethnicity and total: HDL cholesterol ratio.

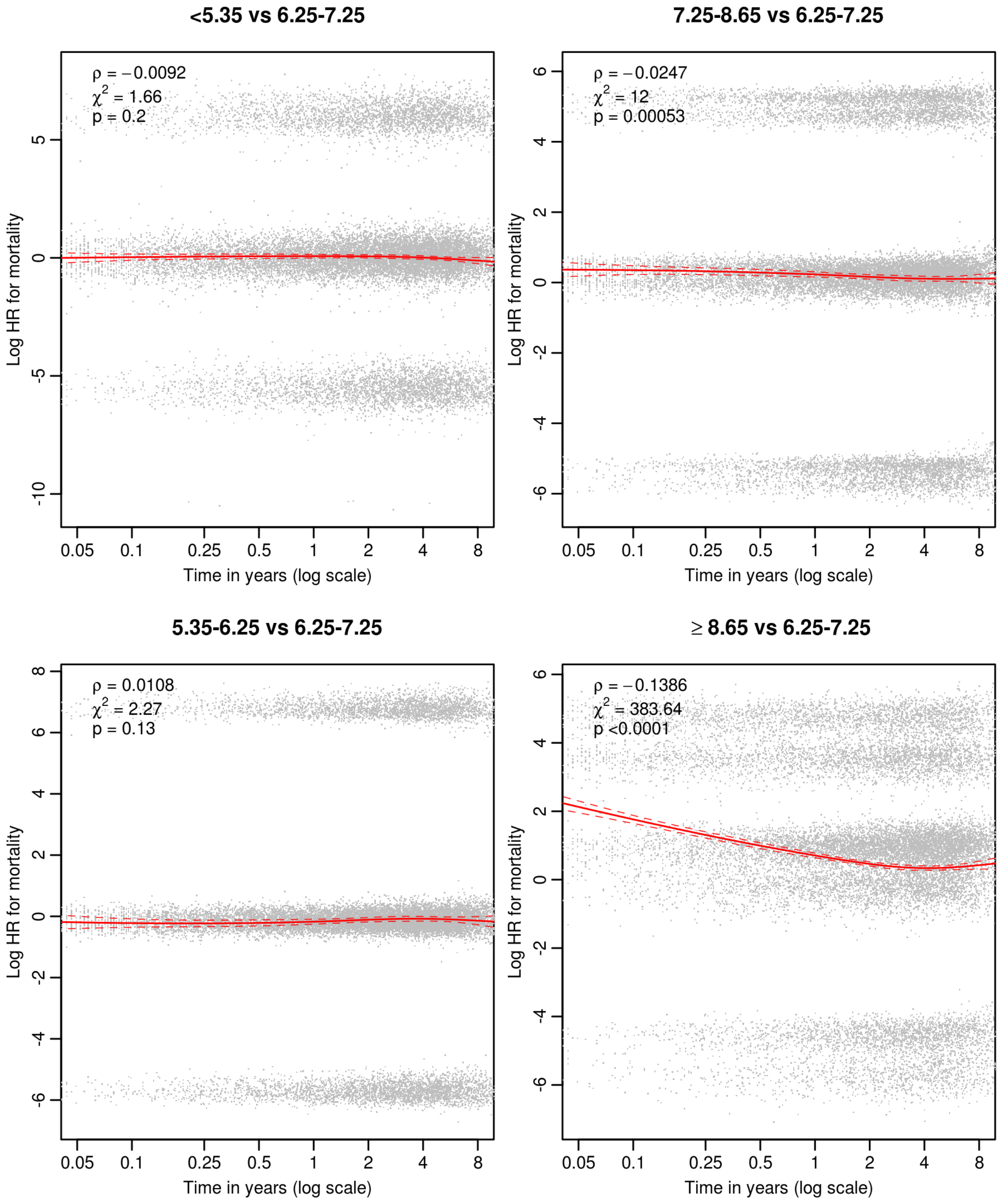

Supplementary Figure S2. Multiply adjusted hazard ratios for all cause mortality by category of total white cell count, by age group: CALIBER

Hazard ratios were adjusted for age, sex, smoking, diabetes, systolic blood pressure, ethnicity and total: HDL cholesterol ratio. P values * < 0.05, ** < 0.01, *** < 0.001

Category of total white cell count		*Hazard ratio (95% CI)*	*P value for interaction (45–60 as reference)*
<2.95	age 30–45	8.18 (5.84, 11.45) ***	0.21
	age 45–60	6.37 (5.12, 7.94) ***	
	age ≥ 60	4.78 (4.00, 5.73) ***	0.038
2.95–5.35	age 30–45	1.23 (1.05, 1.44) **	0.0074
	age 45–60	0.96 (0.88, 1.06)	
	age ≥ 60	0.93 (0.88, 1.00) *	0.56
5.35–6.25	age 30–45	0.97 (0.82, 1.15)	0.14
	age 45–60	0.84 (0.77, 0.93) ***	
	age ≥ 60	0.88 (0.83, 0.94) ***	0.47
6.25–7.25		1 (reference)	
7.25–8.65	age 30–45	1.14 (0.98, 1.33)	0.6
	age 45–60	1.20 (1.10, 1.30) ***	
	age ≥ 60	1.20 (1.14, 1.27) ***	0.93
8.65–10.05	age 30–45	1.19 (1.00, 1.43)	0.15
	age 45–60	1.38 (1.25, 1.53) ***	
	age ≥ 60	1.62 (1.52, 1.73) ***	0.0072
≥ 10.05	age 30–45	1.61 (1.36, 1.90) ***	<0.0001
	age 45–60	2.36 (2.16, 2.58) ***	
	age ≥ 60	2.56 (2.40, 2.72) ***	0.14

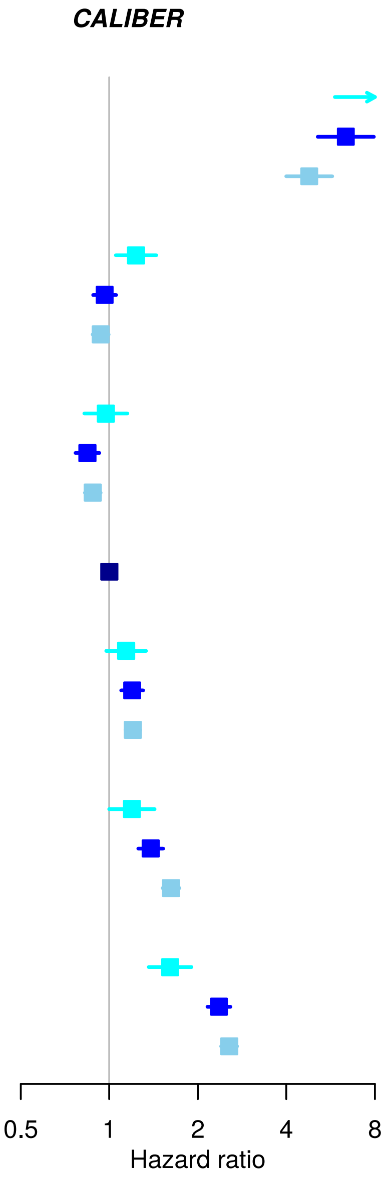

Supplementary Figure S3. Multiply adjusted hazard ratios for all cause mortality by category of total white cell count, by smoking status: CALIBER

Hazard ratios were adjusted for age, sex, smoking, diabetes, systolic blood pressure, ethnicity and total: HDL cholesterol ratio. P values * < 0.05, ** < 0.01, *** < 0.001

Category of total white cell count	*Ex or never smokers* Hazard ratio (95% CI)	*Current smokers* Hazard ratio (95% CI)	P value for interaction
<2.95	5.24 (4.49, 6.11) ***	7.92 (6.06, 10.37) ***	0.0073
2.95–5.35	0.93 (0.88, 0.98) *	1.23 (1.11, 1.37) ***	<0.0001
5.35–6.25	0.86 (0.81, 0.91) ***	0.94 (0.85, 1.03)	0.17
6.25–7.25	1 (reference)	1 (reference)	
7.25–8.65	1.19 (1.12, 1.26) ***	1.19 (1.10, 1.29) ***	0.98
8.65–10.05	1.63 (1.52, 1.75) ***	1.36 (1.25, 1.48) ***	0.0011
≥ 10.05	2.87 (2.68, 3.07) ***	2.02 (1.86, 2.18) ***	<0.0001

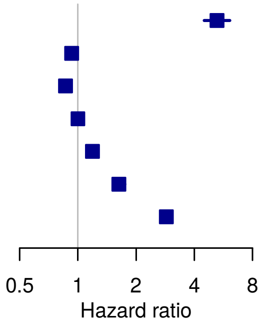

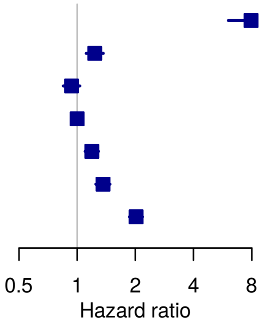

Supplementary Figure S4. Multiply adjusted hazard ratios for all cause mortality by category of total white cell count, by clinical state: CALIBER

Hazard ratios were adjusted for age, sex, smoking, diabetes, systolic blood pressure, ethnicity and total: HDL cholesterol ratio. P values * < 0.05, ** < 0.01, *** < 0.001

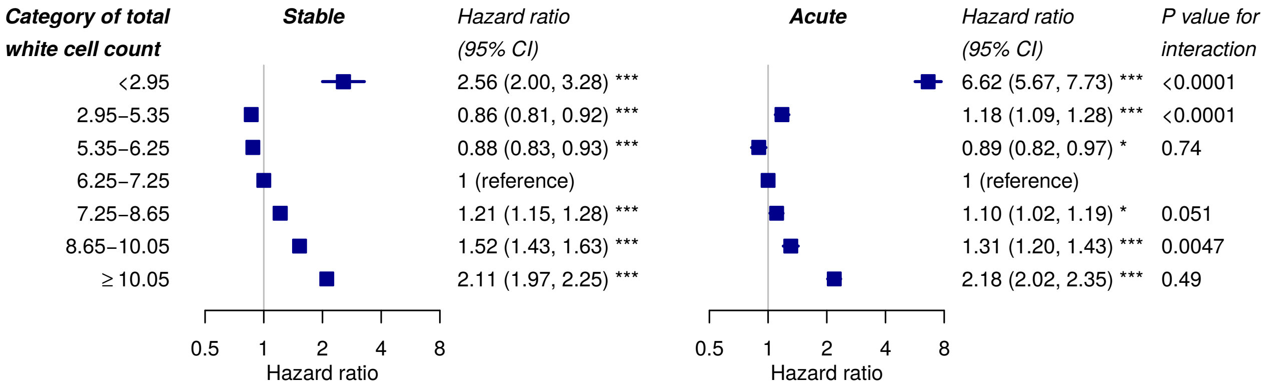

Supplementary Figure S5. Multiply adjusted hazard ratios for all cause mortality by category of total white cell count, by sex: CALIBER

Hazard ratios were adjusted for age, sex, smoking, diabetes, systolic blood pressure, ethnicity and total: HDL cholesterol ratio. P values * < 0.05, ** < 0.01, *** < 0.001

Category of total white cell count	***Men*** Hazard ratio (95% CI)	***Women*** Hazard ratio (95% CI)	*P value for interaction*
<2.95	5.28 (4.28, 6.52) ***	5.79 (4.90, 6.83) ***	0.48
2.95–5.35	1.01 (0.94, 1.08)	0.94 (0.88, 1.01)	0.16
5.35–6.25	0.88 (0.82, 0.94) ***	0.88 (0.82, 0.94) ***	0.98
6.25–7.25	1 (reference)	1 (reference)	
7.25–8.65	1.18 (1.11, 1.26) ***	1.21 (1.14, 1.29) ***	0.56
8.65–10.05	1.59 (1.48, 1.71) ***	1.42 (1.32, 1.53) ***	0.033
≥ 10.05	2.41 (2.25, 2.58) ***	2.36 (2.20, 2.53) ***	0.67

Men

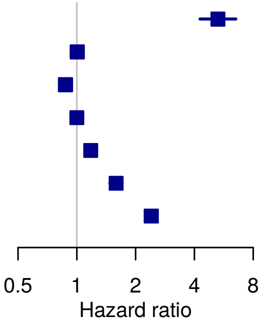

Women

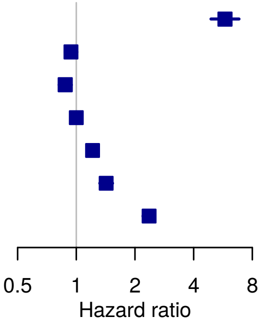

Supplementary Figure S6. Multiply adjusted hazard ratios for all cause mortality by category of total white cell count, by ethnicity: CALIBER

Hazard ratios were adjusted for age, sex, smoking, diabetes, systolic blood pressure, ethnicity and total: HDL cholesterol ratio. P values * < 0.05, ** < 0.01, *** < 0.001

Category of total white cell count		*Hazard ratio (95% CI)*	*P value for interaction (White as reference)*
<2.95	White	5.56 (4.81, 6.42) ***	
	Black	3.49 (1.41, 8.65) **	0.32
	South Asian	8.58 (2.29, 32.09) **	0.52
	Other	12.73 (5.36, 30.21) ***	0.064
2.95−5.35	White	0.97 (0.93, 1.03)	
	Black	0.69 (0.40, 1.20)	0.22
	South Asian	1.05 (0.65, 1.71)	0.75
	Other	1.04 (0.65, 1.65)	0.79
5.35−6.25	White	0.87 (0.83, 0.92) ***	
	Black	0.93 (0.48, 1.81)	0.84
	South Asian	0.97 (0.60, 1.55)	0.67
	Other	0.94 (0.58, 1.52)	0.76
6.25−7.25		1 (reference)	
7.25−8.65	White	1.20 (1.14, 1.25) ***	
	Black	1.18 (0.61, 2.27)	0.97
	South Asian	0.97 (0.62, 1.51)	0.36
	Other	1.49 (0.97, 2.30)	0.31
8.65−10.05	White	1.50 (1.42, 1.59) ***	
	Black	1.78 (0.89, 3.58)	0.63
	South Asian	1.55 (0.93, 2.59)	0.9
	Other	1.51 (0.81, 2.83)	0.99
≥ 10.05	White	2.38 (2.26, 2.50) ***	
	Black	2.51 (1.26, 5.00) **	0.87
	South Asian	2.30 (1.43, 3.69) ***	0.89
	Other	3.00 (1.81, 4.98) ***	0.36

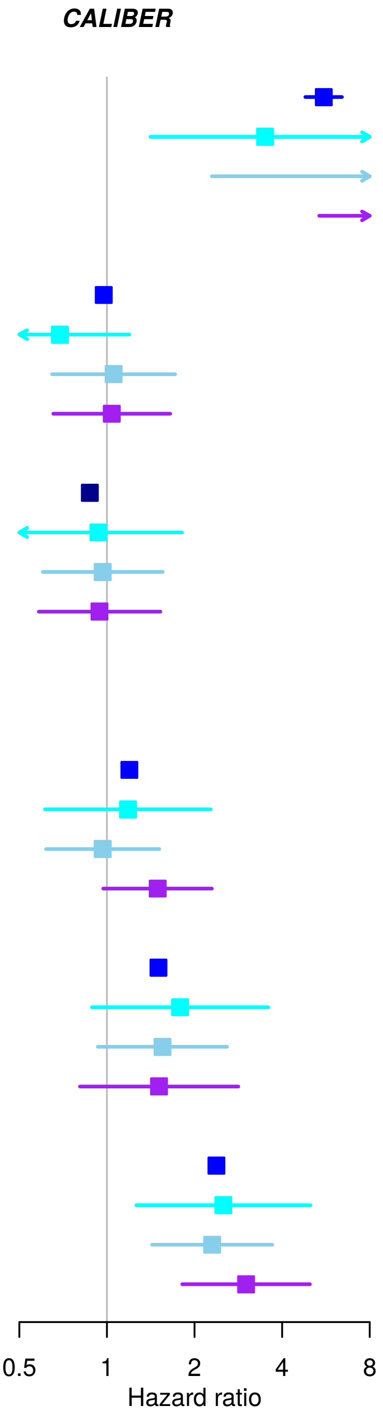

Supplementary Figure S7. Multiply adjusted hazard ratios for all cause mortality by category of total white cell count, by ethnicity: PREDICT

Hazard ratios were adjusted for age, sex, smoking, diabetes, systolic blood pressure, ethnicity and total: HDL cholesterol ratio. P values * < 0.05, ** < 0.01, *** < 0.001. 'Other' group not shown because most categories had too few events for the calculation of hazard ratios

Category of total white cell count		*Hazard ratio (95% CI)*	*P value for interaction (European as reference)*
<2.95	European	6.30 (2.51, 15.79) ***	
	Asian	8.92 (1.14, 69.90) *	0.76
	Indian	–	
	Maori	–	
	Pacific	4.90 (0.68, 35.48)	0.82
2.95–5.35	European	1.03 (0.75, 1.41)	
	Asian	0.92 (0.40, 2.10)	0.79
	Indian	1.56 (0.68, 3.57)	0.35
	Maori	0.94 (0.62, 1.43)	0.73
	Pacific	1.65 (1.11, 2.47) *	0.11
5.35–6.25	European	0.94 (0.71, 1.25)	
	Asian	0.86 (0.36, 2.03)	0.84
	Indian	0.84 (0.30, 2.36)	0.82
	Maori	0.89 (0.61, 1.30)	0.79
	Pacific	1.08 (0.75, 1.56)	0.53
6.25–7.25		1 (reference)	
7.25–8.65	European	1.24 (0.92, 1.67)	
	Asian	1.31 (0.58, 2.97)	0.9
	Indian	0.79 (0.36, 1.73)	0.3
	Maori	1.01 (0.71, 1.44)	0.36
	Pacific	1.13 (0.82, 1.55)	0.67
8.65–10.05	European	1.53 (1.12, 2.08) **	
	Asian	0.99 (0.27, 3.68)	0.52
	Indian	1.03 (0.43, 2.46)	0.38
	Maori	1.22 (0.80, 1.86)	0.32
	Pacific	1.20 (0.82, 1.77)	0.39
≥ 10.05	European	2.59 (1.90, 3.53) ***	
	Asian	1.95 (0.66, 5.79)	0.62
	Indian	1.93 (0.83, 4.51)	0.49
	Maori	1.45 (1.02, 2.08) *	0.0024
	Pacific	1.28 (0.89, 1.84)	0.0045

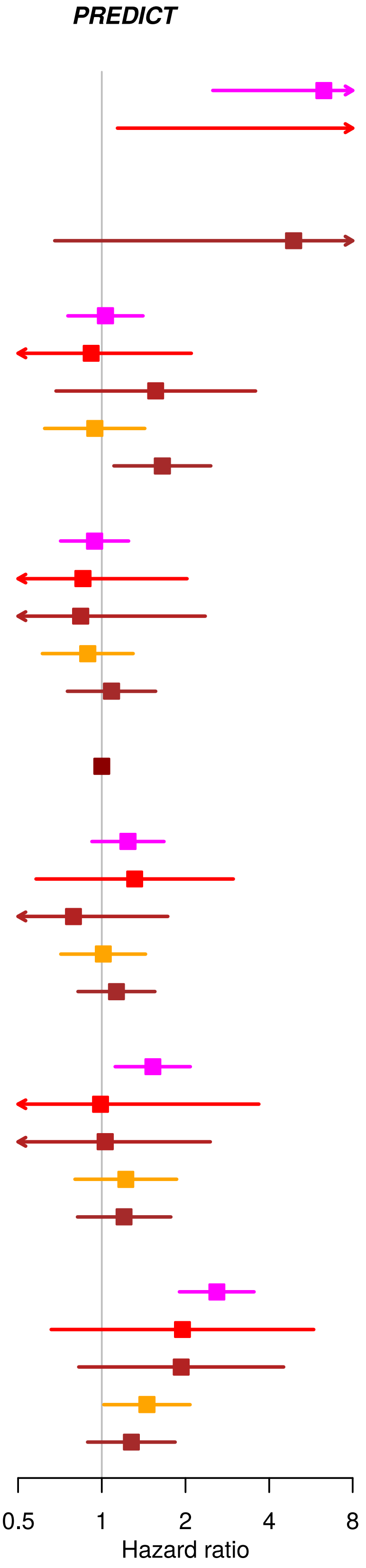

Supplementary Figure S8. Multiply adjusted hazard ratios for all cause mortality by category of total white cell count, by method of multiple imputation: CALIBER

Hazard ratios were adjusted for age, sex, smoking, diabetes, systolic blood pressure, ethnicity and total: HDL cholesterol ratio. P values * < 0.05, ** < 0.01, *** < 0.001

Category of total white cell count	***Normal−based MICE*** *Hazard ratio (95% CI)*	***Random Forest MICE*** *Hazard ratio (95% CI)*
<2.95	5.63 (4.92, 6.45) ***	5.61 (4.89, 6.43) ***
2.95−5.35	0.97 (0.92, 1.02)	0.97 (0.92, 1.02)
5.35−6.25	0.88 (0.83, 0.92) ***	0.88 (0.83, 0.92) ***
6.25−7.25	1 (reference)	1 (reference)
7.25−8.65	1.19 (1.14, 1.25) ***	1.20 (1.14, 1.25) ***
8.65−10.05	1.50 (1.42, 1.58) ***	1.51 (1.43, 1.59) ***
≥ 10.05	2.36 (2.25, 2.48) ***	2.38 (2.27, 2.51) ***

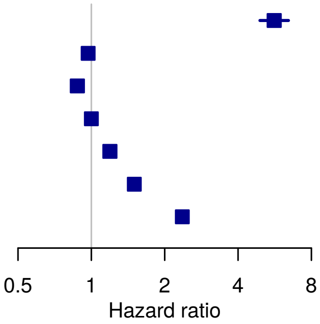

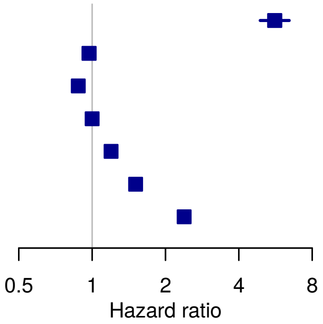

Supplementary Figure S9. Hazard ratios for all cause mortality by category of total white cell count for complete case analysis: PREDICT

Hazard ratios were adjusted for age, sex, smoking, diabetes, systolic blood pressure, ethnicity and total: HDL cholesterol ratio. P values * < 0.05, ** < 0.01, *** < 0.001

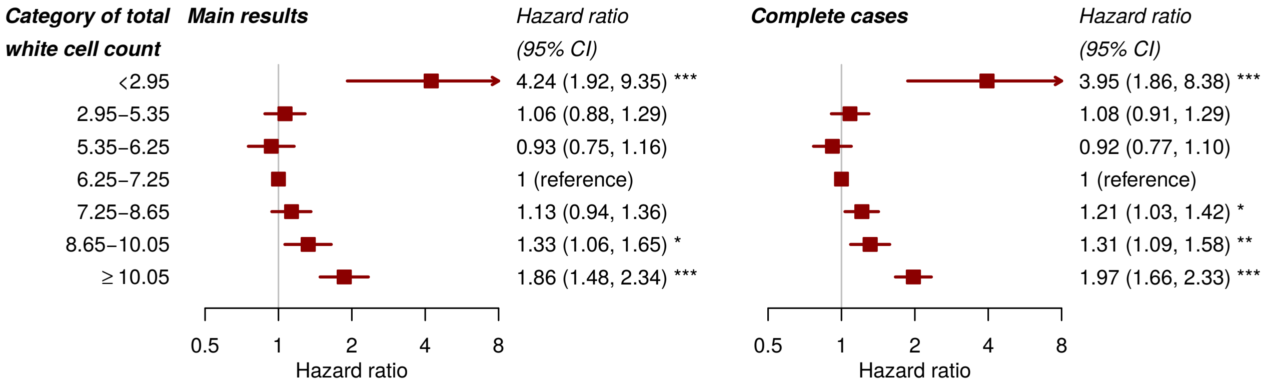

Supplement: supplementary data [file bmjopen-2016-013100supp.pdf]
